# Biofilm Inhibition Activity of Fennel Honey, Fennel Essential Oil and Their Combination

**DOI:** 10.3390/microorganisms12112309

**Published:** 2024-11-13

**Authors:** Lilla Nagy-Radványi, Edit Ormai, Regina Koloh, Virág Diána Ángyán, Béla Kocsis, Erika Bencsik-Kerekes, Péter Szabó, Eszter Csikós, Ágnes Farkas, Györgyi Horváth, Marianna Kocsis, Viktória Lilla Balázs

**Affiliations:** 1 Department of Pharmacognosy, Faculty of Pharmacy, University of Pécs, 7624 Pécs, Hungary; lilla.radvanyi@aok.pte.hu (L.N.-R.); ormai.edit@gytk.pte.hu (E.O.); kolohregina@gmail.com (R.K.); angyanvirag2@gmail.com (V.D.Á.); csikos.eszter@gytk.pte.hu (E.C.); agnes.farkas@aok.pte.hu (Á.F.); horvath.gyorgyi@gytk.pte.hu (G.H.); viktoria.balazs@aok.pte.hu (V.L.B.); 2 Department of Medical Microbiology and Immunology, Medical School, University of Pécs, 7624 Pécs, Hungary; kocsis.bela@pte.hu; 3 Department of Microbiology, Faculty of Science and Informatics, University of Szeged, 6726 Szeged, Hungary; kerekeserika88@gmail.com; 4 Institute of Geography and Earth Sciences, Faculty of Sciences, University of Pécs, 7624 Pécs, Hungary; sz.piiit01@gmail.com; 5 Department of Agricultural Biology, Institute of Biology, University of Pécs, 7624 Pécs, Hungary

**Keywords:** antibiotic resistance, antibiofilm activity, nosocomial infections, disinfectant, natural origin

## Abstract

The eradication of bacterial biofilms remains a persistent challenge in medicine, particularly because an increasing number of biofilms exhibit resistance to conventional antibiotics. This underscores the importance of searching for novel compounds that present antibacterial and biofilm inhibition activity. Various types of honey and essential oil were proven to be effective against a number of biofilm-forming bacterial strains. The current study demonstrated the effectiveness of the relatively unexplored fennel honey (FH), fennel essential oil (FEO), and their combination against biofilm-forming bacterial strains *Pseudomonas aeruginosa*, methicillin-resistant *Staphylococcus aureus*, and *Escherichia coli*, with a series of in vitro experiments. The authenticity of FH and FEO was checked with light microscopy and gas chromatography-mass spectrometry, respectively. Minimum inhibitory concentrations were determined using the microdilution method, and antibiofilm activity was assessed with crystal violet assay. Structural changes in bacterial cells and biofilms, induced by the treatments, were monitored with scanning electron microscopy. FEO and FH inhibited the biofilm formation of each bacterial strain, with FEO being more effective compared to FH. Their combination was the most effective, with inhibitory rates ranging between 87 and 92%, depending on the bacterial strain. The most sensitive bacterium was *E. coli*, while *P. aeruginosa* was the most resistant. These results provide justification for the combined use of honey and essential oil to suppress bacterial biofilms and can serve as a starting point to develop an effective surface disinfectant with natural ingredients.

## 1. Introduction

Despite significant advancements in our understanding of bacterial biofilms, their elimination remains a persistent challenge in medicine. During therapeutic interventions or disinfection of hospital equipment, there exists a constant risk that a single bacterial cell within the biofilm may survive the prescribed duration of treatment, despite the use of high concentrations of antibiotics or disinfectants [1,2]. In immunocompromised patients, bacterial biofilms can manifest throughout various anatomical sites. In addition to the respiratory and digestive systems, microcolonies can also develop in the heart, eyes, or ears [3]. Approximately 65% of bacterial infections and 80% of chronic wound development are due to biofilms, with the incidence of such pathologies continually rising. An increasing number of biofilms exhibit resistance to conventional antibiotics, thereby prolonging healing time and necessitating more invasive interventions. The challenge of eradicating biofilm-forming bacteria also presents a significant obstacle in the design of invasive medical devices and prostheses. Among catheterized and intubated patients, complications are more frequently associated with biofilms [2,4], necessitating the removal of the implicated device (pacemaker, joint prosthesis) if the infection cannot be eradicated, which poses substantial risk. Recent analyses estimate the global economic impact of infections caused by biofilm-forming bacteria at USD 280 billion, underscoring the importance of considering both improved patient outcomes and reduced financial burden when investigating novel therapeutic approaches [5].

Within a biofilm, bacterial cells are embedded in an extracellular matrix composed of various polymeric substances, including exopolysaccharides, proteins, nucleic acids, and lipids. Extracellular polysaccharides often determine the three-dimensional structure of the biofilm and ensure its integrity and cohesion [6]. In these microcommunities, bacterial cells display pleiomorphic behavior, change their morphology, coordinate gene expression, and differentiate metabolomic functions heterogeneously [7]. The effectiveness of the treatment is significantly influenced by whether the antimicrobial agent is able to penetrate the heterogeneously structured biofilm. The production of the extracellular matrix that builds the biofilm is an adaptive mechanism, and its synthesis is induced by the exposure of the bacteria to stress, including the presence of antibiotics and disinfectants. In addition to exopolysaccharides, e-DNA may be another important component of the framework that contributes to the development of resistance [8,9]. In a less-than-ideal case, the spread of the antibiotic/disinfectant slows down and becomes trapped in the biofilm, resulting in the present enzymes neutralizing it [10]. Due to all of this, conventional antibiotic therapy remains a challenge, as the incidence of harmful side effects increases due to the ever-higher doses, so the use of alternative treatments and disinfectants has become relevant. In order to suppress the ever-increasing resistance, it has definitely become necessary to inhibit biofilm formation, understand the underlying mechanisms leading to tolerance, and develop innovative strategies [2].

The biofilm-forming, Gram-negative bacterium *Pseudomonas aeruginosa* can catabolize a wide range of organic molecules for nutrients, making it one of the most versatile and ubiquitous bacteria biochemically. It can survive in many environments, and our body is much more likely to encounter it than other pathogens [11]. In hospitals, it has been isolated from respirators, physical therapy pools, sinks, and cleaning water. Due to its arsenal of virulence factors (e.g., pyocyanin, cytotoxin, proteases, hemolysins, siderophores, exotoxin A, endotoxin, exoenzyme U, exoenzyme S, etc.), *P. aeruginosa* is the cause of many acute and chronic infections. It causes a serious and often life-threatening condition in immunosuppressed patients, and in some cases the mortality rate can reach 40% [12,13,14]. Its biofilm is extremely resistant to antibiotics and disinfectants, and most strains known today are antibiotic resistant [15,16]. Due to these facts, the bacterium *P. aeruginosa* has been classified among the pathogens known as ESKAPE (*Enterococcus faecium*, *Staphylococcus aureus*, *Klebsiella pneumoniae*, *Acinetobacter baumannii*, *P. aeruginosa*, *Enterobacterales* species). The group requires increased attention, as few tools are available to stop the infections they cause, especially in immunocompromised patients [17].

The Gram-positive *Staphylococcus aureus* also belongs to the ESKAPE family and is one of the main causes of hospital-acquired infections; skin and soft tissue, bone, and joint infections; endocarditis; pneumonia and sepsis. In particular, the presence of methicillin-resistant *S. aureus* (MRSA) is still a significant public health problem today, and in the case of immunodeficient, high-risk patients, the proliferation of the bacterium continues to be associated with high morbidity and mortality [18,19]. The MRSA “superbacterium” has been the prototype of multi-resistant nosocomial pathogens for decades, and its biofilm-forming property is the primary means of its outstanding resistance [20]. This bacterium is primarily responsible for catheter infections and infections associated with implanted prostheses and implants. Adhesion to these abiotic surfaces and then the accumulation of bacterial cells in the biofilm promotes the horizontal transfer of genes responsible for resistance (to hospital disinfectants) between cells [18,21].

The Gram-negative bacterium *Escherichia coli* is one of the most common human pathogens and is also capable of producing a significant amount of biofilm, responsible for a wide range of diseases [22]. In immunosuppressed patients or healthy individuals with compromised anatomical and/or physiological barriers, the bacterium can cause severe systemic infections [23]. Based on the clinical symptoms and the type of virulence factor, *E. coli* strains are classified into pathogenic types [24]. Some strains can cause intestinal infections (e.g., diarrheas, among them dysentery caused by Enteroinvasive *E. coli* (EIEC) and various extraintestinal diseases (e.g., urinary and respiratory tract infections, meningitis, or sepsis). The treatment of infections caused by *E. coli* has an annual economic cost of several billion dollars worldwide and is also a serious problem for public health in terms of mortality. This pathogen is also classified as multi-resistant bacteria, as it is resistant to many antibiotics with different mechanisms of action and classified in different classes, as well as several disinfectants [23,25]. The World Health Organization (WHO) also warns that there will be an urgent need for new antimicrobial agents against the mentioned pathogens [26].

The WHO has shifted its approach, now recognizing traditional medicine and natural active ingredients as viable alternatives for treating various diseases and disinfection [27]. Even in developed nations, an increasing number of individuals are opting for natural active ingredients over modern ones, with this trend estimated at 80% in China and India. The antibacterial properties of honey have been demonstrated against both Gram-positive and Gram-negative bacteria, and due to its complex composition of active ingredients, it can be promising in combating certain antibiotic-resistant infections [28,29]. Researchers agree that the main components responsible for the antibacterial effect of honey are hydrogen peroxide (H_2_O_2_), polyphenolic compounds (flavonoids and phenolic acids), and the bee defensin-1 peptide. Manuka honey stands out as the only variety containing large quantities of methylglyoxal (MGO) instead of H_2_O_2_. In addition, the particularly high sugar concentration (causing osmotic stress) and low pH both contribute to antibacterial activity. These properties of different varieties of honey are largely influenced by their botanical and geographical origins, local climatic condition and storage duration [30,31,32]. While fewer data are available on the antibacterial and antibiofilm effect of fennel honey (FH) compared to other honey varieties, its antimicrobial activity has been confirmed by a few research groups [33,34]. The use of plant essential oils dates back to the Middle Ages, when they were employed to protect against epidemics, alleviate fever, headache, and cough, and played a significant role in religious practices [35]. The main component of fennel essential oil (FEO) is anethole, which can comprise 52.5–84.3% of the oil [36]. FEO exhibits antibacterial, analgesic, antispasmodic, circulation-enhancing and anti-inflammatory properties [37]. It appears translucent or yellowish and has an aroma reminiscent of cumin and aniseed. Its antibacterial effects have been observed against numerous pathogens [36,38].

Recent research has revealed that complexity hinders bacteria from developing tolerance, as adapting to multiple active substances is a significantly more protracted and intricate process. Multiple research teams have reported that bacteria remained susceptible to honey and essential oil, with no observed development of resistance [28,29,39,40]. These findings suggest that the combination of these two natural substances may exhibit enhanced efficacy against individual biofilm-forming bacterial strains.

The aim of the current study was to investigate the effectiveness of the relatively unexplored FH and FEO, and their combination, against biofilm-forming bacterial strains (*P. aeruginosa*, MRSA, *E. coli*), performing a series of in vitro experiments. To the best of our knowledge, this is the first study to demonstrate the antibacterial and biofilm inhibition activity of the combination of FH and FEO. Additionally, this research aimed to determine which of the above biofilm-forming bacterial strains was the most susceptible to the anti-biofilm effect of this combination. This research can provide a basis for the development of a surface disinfectant containing substances of natural origin.

## 2. Materials and Methods

### 2.1. Sensory Evaluation and Melissopalynological Analysis of Honey Sample

The fennel honey (FH) used in our study was obtained from a Hungarian beekeeper, and it was harvested in the South Transdanubian region of Hungary in 2022. The sensory analysis included the evaluation of samples in three separate jars. Color was described with standard terminology applied in the case of honeys, ranging from water white to dark amber. Consistency was described as liquid, viscous or semisolid. If any crystallization was observed, its degree and the size of the crystals were mentioned. Odor was characterized as weak, moderate or intense, including specific characters, e.g., reminiscent of a spice. Taste evaluation focused on the degree of sweetness, and if any specific side- or aftertaste (e.g., bitterness) appears. The botanical origin of FH was confirmed by melissopalynological analysis, as described by Koloh et al. (2024) [41].

### 2.2. Analysis of Essential Oil Composition with Gas Chromatography Mass Spectrometry (GC-MS)

Fennel (*Foeniculum vulgare* L.) essential oil (FEO) was a commercial product (doTerra, Europe Ltd., Milton Keynes, UK; identification number: 60204706). For the analysis of the chemical constituents of essential oils, the European Pharmacopoeia (Ph. Eur. 10.0) prescribes gas chromatography [42]. In our study, this instrumental analysis was used, coupled with mass spectrometry [43]. The composition was assessed using retention times and mass spectra for each component, which were then compared to standard values and data from the NIST 2.0 library. Area normalization was applied to calculate the percentages [44]. The parameters of the GC-MS analysis are summarized in Table 1.

### 2.3. Bacterial Strains

In our study, we selected pathogens that form biofilms in humans and play an important role in the development of chronic infections. The antibacterial and antibiofilm activity of the essential oil and honey samples was determined against the Gram-negative *Pseudomonas aeruginosa* ATCC 27853 and *Escherichia coli* ATTC 25922, and the Gram-positive methicillin-resistant *Staphylococcus aureus* (MRSA) ATCC 700698. Pathogens were cultured using Brain Heart Infusion (BHI) Broth (Sigma-Aldrich Ltd., Budapest, Hungary). Bacterial suspensions were incubated in a shaking incubator (C25 Incubator Shaker, New Brunswick Scientific, Edison, NJ, USA) at 37 °C and 60 rpm for 12 h.

### 2.4. Determination of Antibiotic Sensitivity of Test Bacteria

The Kirby-Bauer disk diffusion method was used to determine the antibiotic sensitivity of microorganisms in accordance with the requirements described by CLSI and the Manual of Clinical Microbiology [45,46]. A filter paper disk containing the amount of antibiotics specified by the manufacturer was placed on the surface of the culture medium (Mueller Hinton Agar—Sigma-Aldrich Ltd., Budapest, Hungary) inoculated with the bacterial suspension (equal to the visual turbidity of a no. 0.5 McFarland standard). Then, 15 min after this procedure, the Petri dishes were incubated at 35 ± 2 °C for 16–18 h, and then the inhibition zones were measured under visible light. In the case of MRSA, the 24 h incubation period required for the detection of oxacillin and vancomycin resistance was used. The following antibiotics were tested: amikacin (30 µg), amoxicillin–clavulanic acid (20/10 µg), ceftazidime (10 µg), ceftriaxone (30 µg), cefepime (30 µg), ciprofloxacin (5 µg), erythromycin (15 µg), gentamicin (30 µg), imipenem (10 µg), colistin (10 µg), levofloxacin (5 µg), oxacillin (1 µg), penicillin (1 µg), piperacillin–tazobactam (100/10 µg), trimethoprim–sulfamethoxazole (1.25/23.75 µg), vancomycin (5 µg) (OXOID Ltd., London, United Kingdom) [47].

### 2.5. Determination of Minimum Inhibitory Concentration (MIC)

Minimum inhibitory concentrations of FEO, FH and antibiotics were determined on 96-cell microtiter plates (10^5^ CFU/mL) using the microdilution method according to CLSI guidelines (CLSI Document, 2012) [48]. To prepare stock solutions, the essential oil was dissolved in BHI using 1% Tween 40 emulsifier (Sigma Aldrich Ltd., Budapest, Hungary). Stock solution of FEO was prepared at a concentration of 5 mg/mL. This solution was then subjected to serial two-fold dilutions, lowering the concentration to 0.15625 mg/mL. For honey, stock solutions of 32% and 40% were prepared in BHI, with 32% being used for *E. coli* and *P. aeruginosa*, and 40% for MRSA. Two serial dilutions of the honey were made, leading to final concentrations of 2.0% for *E. coli* and *P. aeruginosa*, and 2.5% for MRSA. Afterward, 100 µL of the tested sample and the bacterial suspension (10^5^ CFU/mL) were each added to each well of a microtiter plate and incubated at 37 °C for 24 h. As a final step to determine the minimum inhibitory concentration, spectrophotometric measurements were performed, where the absorbance values of our samples were determined at 600 nm using a SPECTROstar Nano microplate reader (BMG Labtech, Budapest, Hungary) [46]. As a positive control, gentamicin (Gentamicin Sandoz, solution for injection, 80 mg/2 mL) was used in the case of MRSA and *P. aeruginosa*, while ceftriaxone (Hospira, 250 mg powder, stock solution: 40 mg/mL) was used in the case of *E. coli*. To exclude the effect of Tween40, control tests were also carried out, where the bacterial cells were treated with a BHI solution containing 1% Tween40, without adding essential oil. The minimum inhibitory concentration is the lowest concentration of the test substance that is still effective in inhibiting the growth of the bacterial strain. The lower the value, the more effective the sample. We performed our tests in 6 replicates, searching for the value that inhibited the growth of 90% of the bacteria tested.

### 2.6. Antibiofilm Activity

As a first step, bacterial biofilms were formed on 96-cell microtiter plates. From the bacterial suspension cultures of all three bacteria, prepared at a concentration of 10⁸ CFU/mL, 200 µL was pipetted into the wells of the microtiter plate. The study investigated 24 h bacterial biofilms formed by *P. aeruginosa*, MRSA and *E. coli*. After incubation (24 h at 37 °C), the planktonic cells were washed with physiological saline, and 200 µL of the test sample (FEO, FH, or a combination of FEO and FH) was added to the biofilms. In the study, FEO and FH were applied separately at MIC/2 concentrations, while the FEO-FH combination was applied at MIC/4 concentration. To dissolve the FEO in BHI, 0.01% Tween 40 emulsifier was used. After the treatment (24 h at 37 °C), non-adherent cells were washed with physiological saline. To fix the cells, 200 µL methanol was added to each well and incubated for 15 min at room temperature. To stain the bacterial biofilm, 0.1% crystal violet (CV) solution was added to the cells. CV allows for the measurement of the total biomass of the biofilm by binding to negatively charged surface molecules and polysaccharides in the extracellular matrix of biofilms. After 20 min, the dye remaining in the cells of the microtiter plate was removed with water, and crystal violet was desorbed from the stained biofilm using 200 μL of 33% acetic acid to quantify the results by spectrophotometric measurement. Untreated biofilms were used as a positive control, and pure BHI nutrient solution was used as negative control. In order to investigate the possible inhibitory effect of Tween40 on biofilm formation, we also used an emulsifying control. Absorbance values were determined at 590 nm using a SPECTROstar Nano microplate reader (BMG Labtech, Budapest, Hungary). The biomass of the untreated biofilm as a positive control was considered 100% and compared to the biomass of the bacterial biofilm treated with FEO, FH or a combination of FEO and FH. Eight parallel measurements were carried out [49,50].

Inhibition rates were determined using the formula outlined by Sun et al. (2018) [51]:

Inhibition Rate = (1 − S/C) × 100%

where

- C: represents the average absorbance of the control.
- S: represents the average absorbance of the sample.

### 2.7. Scanning Electron Microscopy (SEM)

SEM images were taken to demonstrate the inhibitory effects of FEO, FH and their combination, to illustrate the structural changes in the bacterial biofilm and the bacterial cells. Previously degreased, sterilized coverslips were placed in 5 mL bacterial suspension (10^8^ CFU/mL) for 4 h at 37 °C. After the incubation period, the coverslips were washed with physiological saline and treated with FH at MIC/2, or FEO at MIC/2, or a combination of these at MIC/4 (5 mL). Untreated coverslips were used as controls. After incubation at 37 °C for 1 day, excess solutions used in the assay were removed and unstained cells were washed with physiological saline. The adherent cells were then fixed with 2.5% glutaraldehyde for 2 h at room temperature. After fixation, samples were dehydrated for 2 × 15 min each at room temperature with a series of increasing concentrations of ethanol solutions: 50%, 70%, 80%, 80%, 80%, 90%, 95%, and 98%. In the next dehydration step, the cover slips were placed in a mixture of tert-butyl alcohol and absolute alcohol in a ratio of 1:2, 1:1, 2:1. In all cases, dehydration was carried out for 1 h at room temperature. The final step of dehydration was performed with absolute tert-butyl alcohol for 2 × 1 h at room temperature. Finally, the coverslips were placed in new t-butyl and allowed to stand for 1 h at 4 °C. The samples were freeze-dried overnight in tert-butyl alcohol. A gold-coated membrane was used to obtain good-quality SEM images. The results were examined using a JEOL JSM IT500-HR scanning electron microscope (Jeol Ltd., Tokyo, Japan) [52].

### 2.8. Statistical Analysis

Statistical analyses were carried out using a Microsoft Excel^®^ 2016 MSO (version 16.0.4266.1001) (Microsoft Corp., Redmond, WA, USA) and the PAST software package version 3.11 [53]. Pairwise comparisons were performed with the Student’s *t*-tests. *p*-values at 1% (*p* ≤ 0.01) were considered significant.

## 3. Results

### 3.1. Melissopalynological and Sensory Analysis

Based on the results of the pollen analysis (Table 2) and the sensory traits, our honey sample can be considered as a monofloral honey. The pollen profile supports that fennel (*Foeniculum vulgare* Mill.) was the source of the dominant pollen. Sunflower (*Helianthus annuus* L.) pollen was observed as a significant minor pollen in the sample. The sensory characteristics of our fennel honey were as follows: bright golden yellow color, viscous with medium dense consistency, intense odor, accompanied by moderately sweet, floral taste with a marked spicy component, reminiscent of anise.

### 3.2. Chemical Composition of FEO

The main component of FEO was trans-anethole (73.1%), while fencone was also identified in a substantial amount in the sample. As minor components, α-terpineol, α-pinene and limonene were detected with percentage values of 3% (Table 3).

### 3.3. Evaluation of Antibiotic Sensitivity of Test Bacteria

The resistance profile of the bacteria included in the study is summarized in Table 4. Based on the results, gentamicin was used for MRSA and *P. aeruginosa*, and ceftriaxone for *E. coli.*

### 3.4. Minimum Inhibitory Concentrations (MICs)

The minimum inhibitory concentrations (MICs) of FEO, FH and the antibiotics used as positive controls were determined by a microdilution assay. The most sensitive pathogen was *E. coli*, since in this case we measured the lowest MIC values, whereas *P. aeruginosa* proved to be the most resistant. Antibiotics were more effective than either FEO or FH (Table 5). The emulsifier used in the case of essential oil (1% Tween40) did not exhibit an antibacterial effect.

### 3.5. Antibiofilm Effect

**Figure 1:**
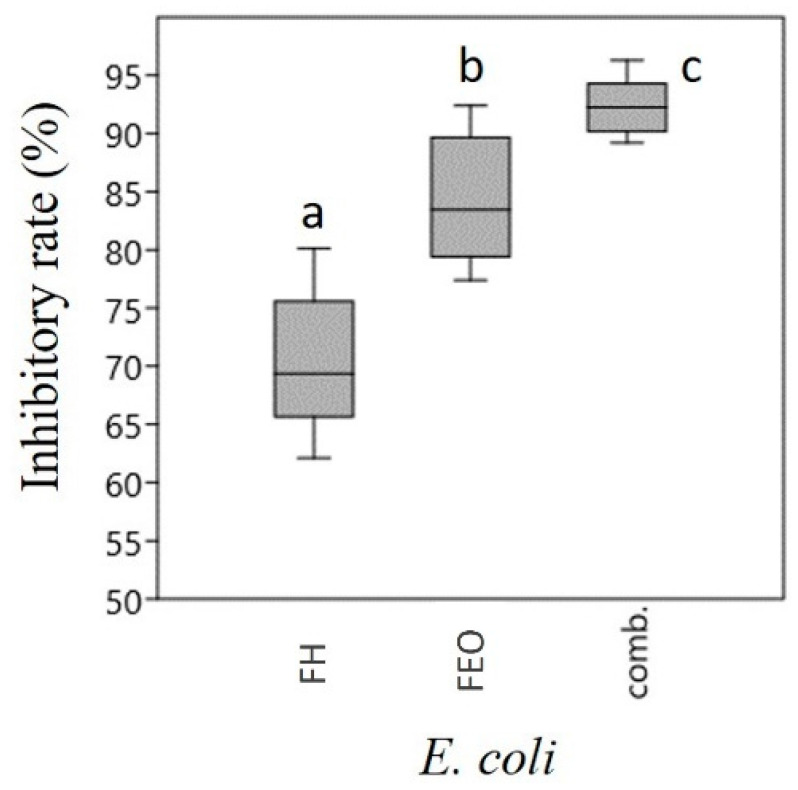

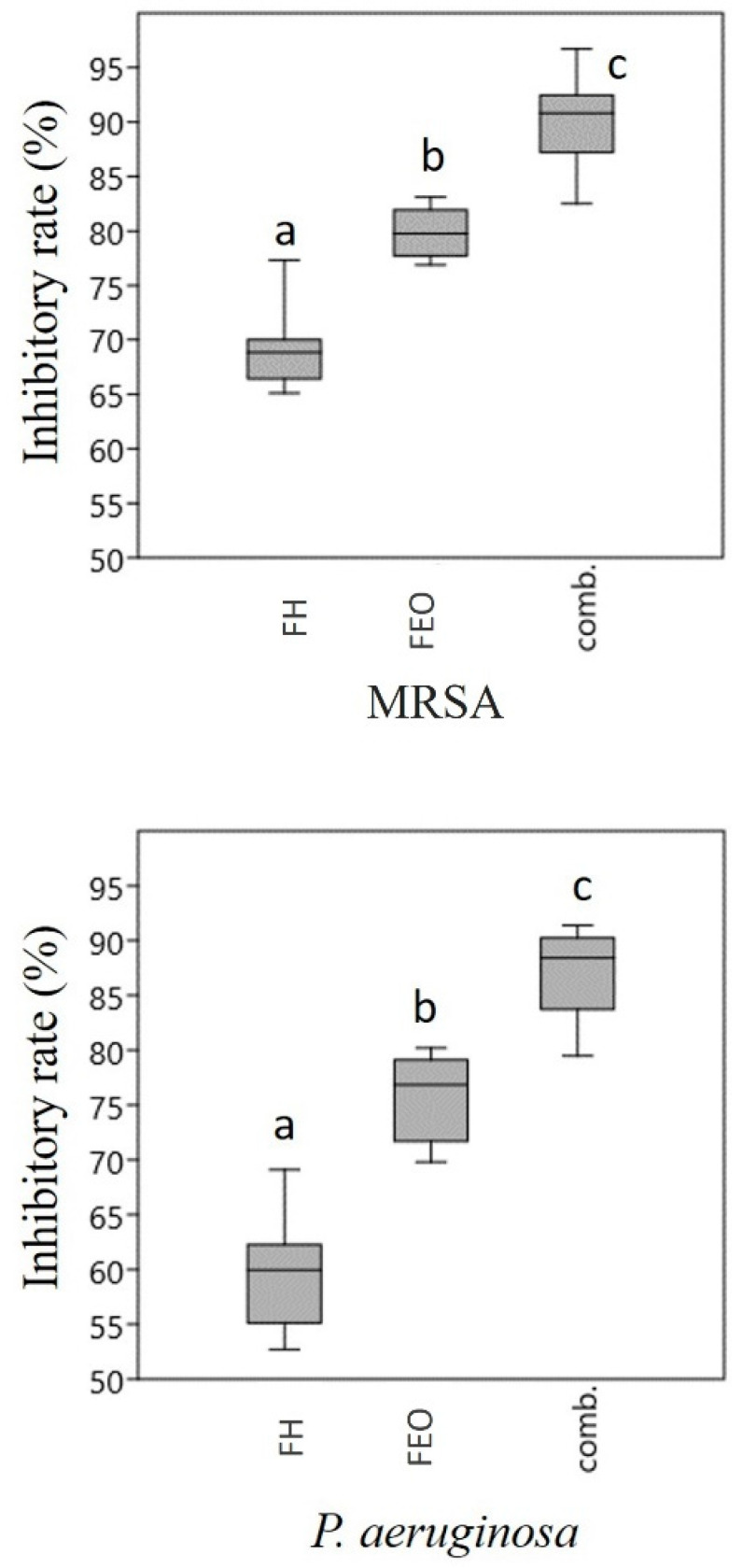
Inhibitory rate of FH, FEO and their combination. Data are expressed using box plots with minimum to maximum values presented by vertical lines, with the median in the plot as a horizontal line. Different lowercase letters above the boxes indicate significant differences between the means of inhibitory rates of FH, FEO and their combination against the given bacteria, according to the Student’s *t*-test (*p* < 0.01), based on results of 8 parallel measurements. *E. coli*: *Escherichia coli*, MRSA: methicillin-resistant *Staphylococcus aureus*, *P. aeruginosa*: *Pseudomonas aeruginosa*. FH: fennel honey, FEO: fennel essential oil, comb.: combination of FH + FEO.

The crystal violet assay clearly demonstrated that both FEO and FH were effective against each bacterial strain, with FEO being more effective compared to FH. The combination of FEO and FH was the most effective, and its inhibition activity reached or exceeded 85.0%, depending on the bacterial strain (Figure 1). The most sensitive bacterium was *E. coli*, whose biofilm formation was inhibited by FH, FEO and the FH-FEO combination by 70.0%, 84.3%, and 92.3%, respectively. Highly similar values were obtained in the case of MRSA, with inhibition rates of 69.1%, 79.8%, and 90.0%, respectively. *P. aeruginosa* was the most resistant bacterial strain, with 59.5%, 75.9%, and 86.9% reductions in the biofilm, respectively. The emulsifier used in the case of essential oil (1% Tween40) did not affect biofilm formation.

### 3.6. Scanning Electron Microscopy (SEM) Images

**Figure 2:**
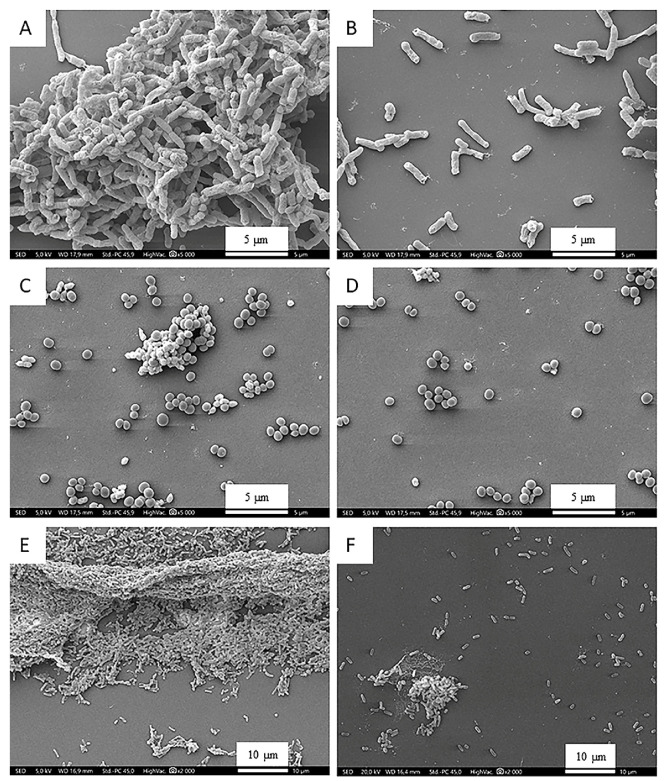
Control ((**A**)—*E. coli*; (**C**)—methicillin-resistant *S. aureus* (MRSA); (**E**)—*P. aeruginosa*) and sample treated with a combination of honey and essential oil ((**B**)—*E. coli*; (**D**)—MRSA; (**F**)—*P. aeruginosa*). In case of taking SEM photos, the following parameters can be observed under each photo: SED, 5.0 kV, WD 17.5 mm, Std.-PC 45.9, HighVac. ×5000 or 2000.

In order to illustrate the structural aspects of our results, SEM was applied (Figure 2). Since the combination of FEO and FH proved to be the most effective, SEM images were taken following combination treatments. The continuous, mature biofilm formed by the bacterial strains included in this study is clearly visible in the images of the untreated samples (Figure 2A,C,E). In the case of the treated samples, the formation of the biofilm did not occur, since the bacterial cells present are scattered (Figure 2B,D,F). It should be emphasized that *P. aeruginosa* (Figure 2E,F) proved to be the most resistant bacterium. The SEM image shows that despite the treatment, a smaller biofilm unit was formed. In contrast, following the treatment, either *E. coli* (Figure 2A,B) or MRSA (Figure 2C,D) bacteria were no longer aggregated, but could be observed in planktonic form.

## 4. Discussion

The growing concern of antibiotic resistance has intensified the need to discover novel, alternative therapeutic approaches. Kwiatkowski and his research team (2017) also realized that the increase in the number of staphylococcal infections and carriers among healthcare workers is prompting us to look for more and more effective antibacterial agents [55]. In light of this, their investigation was aimed at testing the effectiveness of FEO against the pathogen *S. aureus*. When FEO was used together with cefoxitin, mupirocin, co-trimoxazole and ciprofloxacin, the size of the inhibition zones increased significantly compared to the antibiotic control used alone. Their research showed that FEO in combination with mupirocin may be considered as a natural alternative in the eradication of *S. aureus* phenotypes with methicillin and/or macrolide–lincosamide–streptogramin B resistance and may be able to decrease the growth rate of antibiotic resistance. Alam’s research team (2022) reported that FEO in nanogel form was effective against *S. aureus* [56]. Naaz and his colleagues (2022) investigated FEO in terms of both antioxidant and antibacterial effects, including the same bacterium species (*E. coli*, *P. aeruginosa* and *S. aureus*) as in our study, in addition to *Bacillus subtilis* [57]. The somewhat controversial disk diffusion technique was employed to demonstrate that FEO exhibits efficacy against both Gram-negative and Gram-positive pathogens. The experimental setup revealed that *P. aeruginosa* displayed the highest resistance to FEO, a finding that resonates with our observed results.

Essential oils can both inhibit bacterial growth (bacteriostatic) and kill bacteria (bactericidal) [58]. Different essential oils have different antibacterial effects. Bouyahya et al. (2017) tested the antibacterial effect of *Mentha pulegium* and *Rosmarinus officinalis* essential oils [59]. Comparing their activity, mint essential oil proved to be more effective against *S. aureus*, *P. aeruginosa*, *Listeria monocytogenes*, *B. subtilis*, *E. coli,* and *Proteus mirabilis.* The essential oil of thyme (*Thymus*) species, known for its antibacterial effect, exerted an inhibitory effect against both Gram-negative and Gram-positive bacteria [60]. Pesavento et al. (2015) compared the antibacterial effect of the essential oils of rosemary, cinnamon bark, thyme, sage and oregano using the agar hole diffusion method [61]. Their study revealed that thyme essential oil proved to be the most effective, and it was established that sage essential oil had no antibacterial effect against *L. monocytogenes*, *S. aureus*, *Salmonella enteritidis* and *Campylobacter jejuni*. In addition, it is important to emphasize that the essential oil of oregano proved to be one of the most effective agents against *S. Typhimurium*, *E. coli*, *B. cereus*, *P. aeruginosa* and *Enterococcus faecalis* microorganisms [62]. The essential oil of cinnamon bark and mustard individually inhibited *S. aureus*, *B. cereus*, *E. coli*, *P. aeruginosa*, *P. fluorescens*, *P. putida*, *P. carotovorum* and *S. enterica* bacteria [63]. There are data on essential oil mixture testing, not only with mustard essential oil. The essential oils of cinnamon bark and cloves showed a synergistic effect, since their combined use proved to be more effective against *S. aureus*, *L. monocytogenes*, *P. aeruginosa* and S. Typhimurium [64]. Although lavender essential oil is best known in the cosmetics industry, it has a significant antibacterial effect against *E. coli*, *S. aureus*, *P. aeruginosa*, *L. innocua*, *L. monocytogenes*, *S. aureus*, *B. subtilis*, *P. vulgaris* bacteria [65]. The inherent potential in essential oils’ antibacterial effect is indisputable [66], so it is essential to investigate the inhibition of additional bacteria with essential oils. Studies have shown that phenylpropane derivatives and phenolic components in essential oils have potent antibacterial properties [67]. These components primarily target bacterial cell membranes, affecting their permeability and disrupting ion transport (K^+^, Ca^2+^, Na^+^). They also cause protein denaturation [68] and inhibit acetic enzyme processes [69]. Bacterial death occurs due to changes in membrane permeability, ion transport disruption, mitochondrial process inhibition, and ATP balance disturbance [66]. The main component of FEO is trans-anethole. Research conducted by Medeiros and colleagues (2023) revealed that trans-anethole exhibits bactericidal properties against certain *Enterococcus* strains [70]. It is crucial to emphasize that in essential oils, the presence of minor components also contributes to the antibacterial and anti-biofilm formation effect of the oil. This is further evidenced by Donati et al.‘s (2014) study, which did not yield promising results regarding trans-anethole’s antibacterial impact on *S. aureus*, *P. aeruginosa* and *E. coli* [71]. Nevertheless, essential oil with a complex composition demonstrates effectiveness. In addition, Hançer’s (2018) research indicated significant anti-quorum sensing (QS) activity of trans-anethole against the pathogen *P. aeruginosa* [72]. Mirzaei’s research group (2022) also confirmed trans-anethole’s anti-QS activity against the bacterium *P. mirabilis*, with their study also examining its ability to inhibit biofilm formation [73]. The specific roles of major and minor components in FEO’s antibacterial effect have not yet been fully elucidated.

Scientists agree that honey’s antibacterial effect stems from hydrogen peroxide (H_2_O_2_), polyphenolic compounds (flavonoids and phenolic acids), and the bee defensin-1 peptide [31,32]. Its high sugar concentration, leading to osmotic stress, and low pH also contribute to this effect. The antibacterial properties of different honey types depend on their botanical and geographical origin, regional climate, and storage duration [30,32]. Oxygen produced during honey hydrolysis can interact with polyphenols, accelerating their autoxidation. These compounds then act as prooxidants, damaging bacterial genetic material. Reactive oxygen species (ROS) from autooxidation reactions can impede bacterial growth by degrading bacterial DNA. Honey’s acidity affects membrane proteins, changing permeability, leading to essential ion (e.g., potassium ion) leakage and bacterial cell death. Researchers have identified antimicrobial peptides in bees that protect bee colonies. These polymers can penetrate bacterial membranes and disrupt protein folding. The defensin-1 peptide produced in the salivary gland of the bee, which is incorporated into honey during nectar processing, increases the permeability of the bacterial membrane or, passing through the membrane, inhibits intracellular targets [74]. Additionally, the osmotic effect of honey’s high sugar concentration contributes to its antimicrobial activity when not significantly diluted.

To date, limited research has been conducted on the combined efficacy of honey and essential oil in combating bacteria, and these studies do not address the inhibition of biofilm formation. Imtara et al. (2018) evaluated the interactions between 12 honey varieties and oregano (*Origanum vulgare*) essential oil, as well as their joint impact on six bacterial strains (including three *E. coli* strains, *P. aeruginosa*, *S. aureus* and *Streptococcus faecalis*), using the checkerboard assay [75]. Approximately 42% of the tested honey–essential oil combinations acted in a synergistic or additive manner, while 58% of the combinations had no interaction or no effect on the bacterial strains. In the case of effective combinations, the MIC values decreased by 1–4-fold and 1–8-fold against Gram-positive and Gram-negative bacteria, respectively. Assaggaf et al. (2022) investigated the effectiveness of eucalyptus honey, eucalyptus essential oil, and their combined form against the pathogens *E. coli*, *P. mirabilis*, *Salmonella* Typhimurium, *B. subtilis*, *S. saureus*, and *L. monocytogenes*, applying the disk diffusion method [76]. Their findings revealed that eucalyptus essential oil was more potent than both eucalyptus honey and the mixture of oil and honey. This outcome contrasts with our research, which found the combination to be the most effective, compared to honey or essential oil alone. The discrepancy may be attributed to the different experimental techniques employed in each study. It is worth noting that the disk diffusion method has limitations when used with essential oils, as not all components can effectively diffuse into the agar medium.

Our investigation evaluated the efficacy of FEO, FH, and their combined use against pathogens capable of causing hospital-acquired infections. It should be noted that our research group was the first to identify the antibacterial and biofilm-inhibiting properties of FH, while also verifying the in vitro antibacterial and anti-biofilm effects of FEO. We demonstrated that the joint application of FH and FEO most effectively inhibited the biofilm formation of three bacterial strains potentially involved in nosocomial infections. The increased effectiveness of the FEO-FH combination is due to the fact that both the oil and the honey exert their effect on several points of action, as shown above. Our findings, together with results obtained by other research groups performing in vitro assays to test the antibacterial efficacy of FEO and FH, can provide a basis for further studies aimed at developing an efficient, natural, multi-target combination surface disinfectant preparation that exhibits antibacterial and biofilm-inhibiting properties against pathogens implicated in nosocomial infections.

## 5. Conclusions

As a result of the ever-increasing prevalence of antibiotic resistance, it is absolutely necessary to examine substances of natural origin that can be potentially effective during complementary and preventive therapy. Until now, we had no data on the effectiveness of fennel honey and fennel essential oil and their combinations. Using in vitro microbiological assays, we demonstrated the antibacterial and biofilm-inhibiting effect of fennel honey and essential oil individually, as well as in combination. We confirmed that the combined use of two natural substances (honey and essential oil) was effective against the most common pathogens causing problems in nosocomial infections (*P. aeruginosa*, *E. coli*, MRSA). Our results can serve as a basis for further formulation tests, which additionally enhance efficient application, even in the case of developing surface disinfectant preparations with natural ingredients that prevent biofilm formation.

## Figures and Tables

**Table 1:** Conditions of GC-MS analysis.

	GC-MS
Device	Agilent 6890N (Agilent Technologies, Santa Clara, CA, USA)
Column	30 × 0.25 mm i.d, Agilent SLB-5MS (film thickness 0.25 µm)
Program	60 °C 3 min, 8 °C/min 60–250 °C, 250 °C 1 min
Carrier gas	High-purity helium 6.0, 1.0 mL/min, (37 cm/s), constant flow
Injector	250 °C
Injection	Split ratio 1:50
Detector	Agilent 5973N (MS)

**Table 2:** Pollen spectrum of sweet fennel honey.

Pollen Type—Relative Frequency (%)					
FH	*Foeniculum vulgare*	*Helianthus annuus*	*Tilia* sp.	*Brassica napus*	Other
	62	13	7	4	14

**Table 3:** Composition of FEO, determined with GC-MS (n = 3).

Compounds	KI	tR (min)	Percentage of Compounds (%)
α-Pinene	923	5.1	3.9
Camphene	940	5.5	0.3
β-Pinene	968	6.1	0.3
β-Myrcene	978	6.3	0.7
α-Phellandrene	996	6.7	0.6
*p*-Cymol	1015	7.1	0.2
Limonene	1020	7.2	3.0
γ-Terpinene	1050	7.9	0.8
**Fencone**	**1083**	**8.6**	**11.5**
Camphor	1141	9.9	0.3
α-Terpineol	1191	10.9	4.5
4-Anisaldehyde	1255	12.1	0.4
**trans-Anethole**	**1292**	**12.9**	**73.1**
Anise ketone	1378	14.4	0.3
not identified			0.1
Total			100%

KI: Kovats index [54]. Percentage values of compounds are presented as means of three parallel measurements. Standard errors of means were between 0.01 and 0.05.

**Table 4:** Examination of antibiotic sensitivity of test bacteria by disk diffusion.

Antibiotics	MRSA	*Pseudomonas aeruoginosa*	*Escherichia coli*
Amikacin	-	S	S
Amoxicillin–clavulanic acid	-	-	S
Ciprofloxacin	R	S	S
Ceftazidime	-	S	S
Cefepime	-	S	S
Ceftriaxone	-	-	S
Erythromycin	-	-	S
Gentamicin	S	S	S
Imipenem	-	S	S
Colistin	-	S	S
Oxacillin	R	-	S
Levofloxacin	-	S	S
Penicillin	R	-	S
Piperacillin–tazobactam	-	S	S
Trimethoprim–sulfamethoxazole	-	R	S
Vancomycin	S	-	S

R: resistant, S: sensitive; MRSA: methicillin-resistant *Staphylococcus aureus.* “-” means no data.

**Table 5:** The minimum inhibitory concentrations (MICs) of FH, FEO and the control antibiotic against each bacterial strain.

	MIC Values (mg/mL)		
	*E. coli*	MRSA	*P. aeruginosa*
Fennel Honey	86.960	111.111	190.480
Fennel Essential Oil	0.313	0.625	1.250
Antibiotics *	0.004	0.013	0.006

* Antibiotics were used as control: ceftriaxone in case of *E. coli* (*Escherichia coli*); gentamicin in cases of MRSA (methicillin-resistant *Staphylococcus aureus*) and *P. aeruginosa* (*Pseudomonas aeruginosa*).

## Data Availability

The original contributions presented in this study are included in the article. Further inquiries can be directed to the corresponding author.
